# Fall detection using accelerometer-based smartphones: Where do we go from here?

**DOI:** 10.3389/fpubh.2022.996021

**Published:** 2022-10-17

**Authors:** Tristan Stampfler, Mohamed Elgendi, Richard Ribon Fletcher, Carlo Menon

**Affiliations:** ^1^Biomedical and Mobile Health Technology Laboratory, Department of Health Sciences and Technology, ETH Zurich, Zurich, Switzerland; ^2^Mobile Technology Group, Department of Mechanical Engineering, MIT, Cambridge, MA, United States

**Keywords:** remote patient monitoring, mHealth, mobile health, daily activity detection, behavioral tracking, digital phenotyping, wearable devices, smart assistive technology

## Abstract

According to World Health Organization statistics, falls are the second leading cause of unintentional injury deaths worldwide. With older people being particularly vulnerable, detecting, and reporting falls have been the focus of numerous health technology studies. We screened 267 studies and selected 15 that detailed pervasive fall detection and alerting apps that used smartphone accelerometers. The fall datasets used for the analyses included between 4 and 38 participants and contained data from young and old subjects, with the recorded falls performed exclusively by young subjects. Threshold-based detection was implemented in six cases, while machine learning approaches were implemented in the other nine, including decision trees, k-nearest neighbors, boosting, and neural networks. All methods could ultimately achieve real-time detection, with reported sensitivities ranging from 60.4 to 99.3% and specificities from 74.6 to 100.0%. However, the studies had limitations in their experimental set-ups or considered a restricted scope of daily activities—not always representative of daily life—with which to define falls during the development of their algorithms. Finally, the studies omitted some aspects of data science methodology, such as proper test sets for results evaluation, putting into question whether reported results would correspond to real-world performance. The two primary outcomes of our review are: a ranking of selected articles based on bias risk and a set of 12 impactful and actionable recommendations for future work in fall detection.

## Introduction

Falls are the second leading cause of unintentional injury deaths worldwide, according to the World Health Organization (1). More than 37 million falls each year are severe enough to require medical attention (1), and people over 60 years old are the most concerned with fatal falls (2). The global population of over-60s was estimated to be 962 million in 2017, a number which is expected to double by 2050 (3).

**Figure 1:**
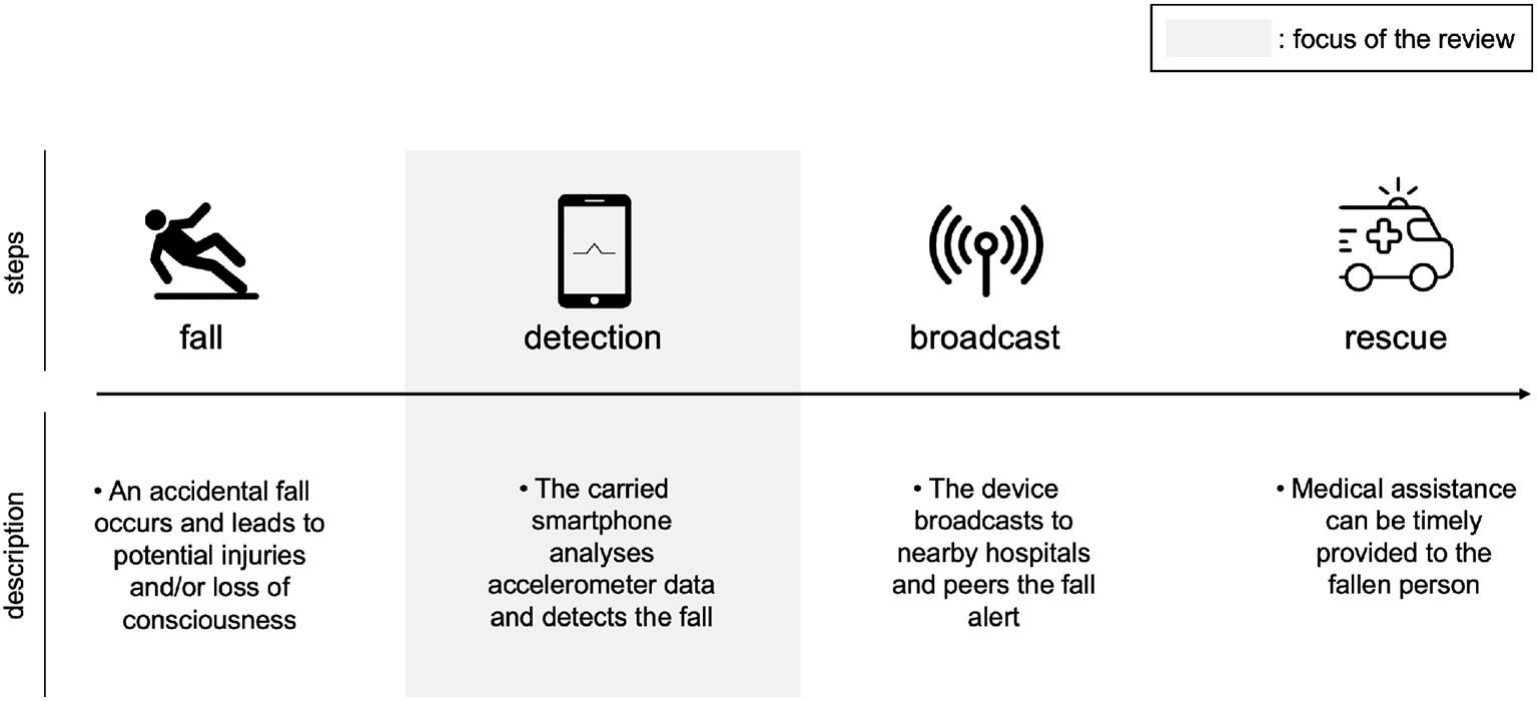
Principle for fall detection with a smartphone, our review focuses on the detection step.

Furthermore, this population increasingly lives in isolated conditions, making falls even more dangerous because immediate assistance cannot always be provided (2). Other populations concerned with falls are younger people with disabilities or people recovering from an operation or an injury. There is thus a need for technologies to better handle falls, such as falling detection systems that can automatically send alerts. The core principle of phone-based fall detection is illustrated in Figure 1.

We offer here a review of research studies (4–18) that propose practical methods to detect falls using a pervasive device that most people carry every day: smartphones. These devices have many built-in sensors, including an accelerometer, which can capture the acceleration caused by a fall. Data can be collected continuously during the day, which can then be analyzed using threshold-based algorithms or more modern machine learning techniques. This raises the possibility of real-time operation, in which the chosen methodology would be expected to achieve real-time detection and alerting. The selected articles each report the building of an app that assumes a phone being kept in a common location, like in a pocket. The development of such fall apps presents four main challenges: to design an experimental set-up and build an appropriate dataset for fall recognition; to conceive and tune an algorithm; to evaluate the results, including defining relevant metrics and an evaluation procedure; and to ensure that the detection and all associated functionalities work well-together.

The choice of using a phone to carry such monitoring is part of the broader concept of “digital phenotyping,” which is about tracking people's behavior using data from personal digital devices (19). Smartphones bring together virtually all characteristics one can wish to have for an efficient monitoring: a huge adoption rate, a multitude of sensors, computing power and network connection. These make them especially relevant for fall detection but also other tasks such as general gait analysis or mental health assessment. Van Laerhoven et al. (20) determined that users have their smartphones on them on average 36% of daytime. The main pattern they describe is that while sitting, users tend to put their phone on the table and place it in their pockets when they move, which suits well a phone-based fall detection paradigm discussed here. The trousers' pocket emplacement is the first to carry a smartphone when on the body for both men (21) and women (22). However, there is also a significant gender difference as carrying a phone separately in a bag is also very popular among women (22) and only mildly adopted among men (21).

This review aims to provide a detailed overview of existing solutions reported as working for fall detection using smartphone accelerometers. We describe the various approaches, highlight best practices, and note areas for improvement, presenting a review that will give valuable insights for future research into fall detection and offer a significant head-start for future development.

## Related work and technologies

First we would like to shortly mention another review work from Casilari et al. (23), its main conclusions but also limitations regarding fall detection with a smartphone. The study presents quite extensively the technological and IT possibilities for a fall detection system. However from our perspective, the most relevant insights from this study lie on its critique on evaluation methods being performed to assess a solution performance. It notably points out the lack of common data across studies to draw comparisons, the heterogeneity of fall typologies used and the lack of real world evaluation. Nevertheless, this review clearly has non negligible limitations. From a timeline perspective it was published in 2015 which means it could not capture most recent papers and development of the topic. This is particularly illustrated by a lack of discussion on machine learning choices and issues one must face when developing a time series classifier for fall detection.

Second, we also would like to indicate alternative technologies that have been explored to detect falls beyond using the smartphone. It is possible to group them into two separate categories: wearable sensors and external sensors. The first category entails wearing on the body a sensor (commonly an accelerometer), which is usually built in a smartwatch or a belt. Some commercial application have even seen the day of light with for instance the Apple Watch offering a fall detection feature to its users. The second category corresponds generally to devices that can be found in a smart-home and that can monitor their environment. The list includes radars, infrared sensors, ultrasounds, or even image processing of camera recordings. For further reference on technological possibilities for fall detection, we recommend the review from Wang et al. (24).

## Materials and methods

### Search strategy

To conduct our review, we searched three research databases-*IEEE Xplore, PubMed*, and *Embase*-chosen for their relevance to this topic at the nexus of health and modern data analysis. We used their advanced keyword search functionalities to find articles published in the last decade (from January 1st, 2012, to the date of our search on March 17th, 2022). Our search request contained the following search terms: (fall OR falling) AND (accelerometer) AND [smartphone OR (smart phone)].

### Inclusion and exclusion criteria

To refine the initial search results, we applied the following additional criteria based on content. **Screening**: the article must be readable by the reviewers. **Type**: the article should be a primary research study. **Goal**: the study should be focused on fall detection. **Device**: the employed device should be a smartphone with an accelerometer. **Phone positioning**: the phone should be positioned in a natural and common way without additional straps. **App**: the research should have led to the development of an app as a proof-of-concept.

## Results

**Figure 2:**
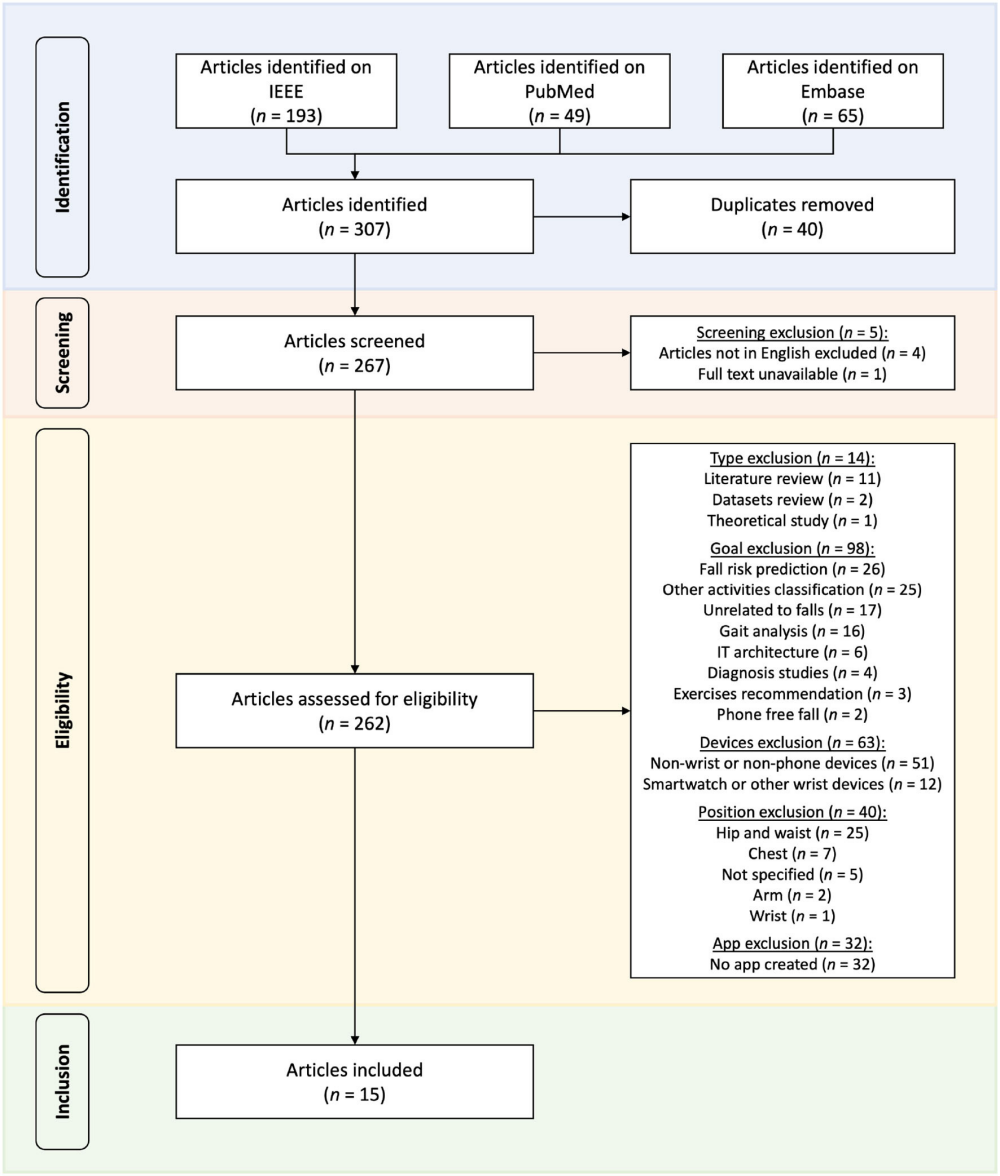
PRISMA diagram of the selection of relevant articles for practical fall detection using smartphone accelerometers.

Applying these criteria as filters, we reduced our initial results set of 267 articles to the 15 most relevant to understand the current state of the art. The complete process can be visualized with the PRISMA diagram in Figure 2.

In this section, we aim to provide a comprehensive comparative view of the selected articles by analyzing them according to a set of features and metrics. Table 1 summarizes all information collected on the studies. After a careful reading of each article, we collated their content across the following dimensions:

- **Publication:** general information on the publication to provide context
- **Data collection:** characteristics of experimental set-up characteristics to understand how the data were collected
- **Scope:** the range and the depth of the investigation
- **Data analysis:** techniques used to illustrate the design of the proposed algorithms
- **Evaluation:** metrics reported to compare performances

**Table 1:** Summary table of the 15 selected articles.

**Publication**		**Scope**			**Data collection**								**Data analysis**			**Evaluation**				
**References**	**Year**	**No. ADLs**	**No.** **Falls**	**Phone drop**	**Data- set** ^a^	**No. Part**	**Age**	**Gender (M,F)**	**Device position** ^b^	**Sensors** ^c^	**Sampl. freq.**	**Falls reps**	**Main features** ^d^	**Filters** ^e^	**Algorithm**	**Sensi- tivity**	**Speci- tivity**	**Accu- racy**	**Eval. method**	**Power check**
Shi et al. (4)	2012	5	5	Yes	O	4	20–26	3, 1	TP	A	50 Hz	100	GR, MA, TI	LP	SVM	90.0	95.7	N/A	LOSO	No
Aguiar et al. (5)	2014	10	10	No	O	36	22–69	28, 8	TP	A	67 Hz	1,025	SF,MA	LP	Dec. Tree	94.0	90.2	92.0	CV	Yes
Medrano et al. (6)	2014	N/A	8	No	O	10	20–42	7, 3	TP,BA	A	50 Hz	240	N/A	N/A	kNN	92.0	95.0	N/A	CV	No
Pierleoni et al. (7)	2015	10	10	No	O	5	24–29	N/A	TP,SP	A,M	40 Hz	150	SF	LP	SVM	99.3	96.0	97.7	Train set	No
Helmy and Helmy (8)	2015	N/A	4	Yes	O	14	N/A	N/A	TP,OH	A	20 Hz	400	TI,MA	N/A	Thresholds	95.0	90.0	N/A	Train set	Yes
Cao et al. (9)	2015	N/A	17	No	O	20	N/A	N/A	TP	A	17 Hz	648	MA	N/A	Boosting	88.0	74.6	N/A	Train set	Yes
Vermeulen et al. (10)	2015	16	10	No	O	8	18–24	4, 4	TP,BE	A	67 Hz	400	MA	N/A	Thresholds	90.0	87.0	N/A	Train set	No
Srisuphab et al. (11)	2016	N/A	9	No	O	N/A	N/A	N/A	TP	A	N/A	90	TI,MA,VE	N/A	Thresholds	95.6	N/A	N/A	Train set	No
Chaitep and Chawachat (12)	2017	2	8	Yes	O	N/A	N/A	N/A	TP	A	N/A	800	MA,GF	N/A	Thresholds	83.0	91.5	N/A	Train set	No
Tsinganos and Skodras (13)	2017	12	4	No	P	24	22–47	17, 7	TP	A	50 Hz	288	TD,SF	LP	kNN	97.5	94.9	N/A	CV	Yes
Tran et al. (14)	2017	3	1	No	O	15	N/A	N/A	TP,BE	A	50 Hz	86	MA,TD	N/A	Perceptron	60.4	94.8	82.5	Val. set	No
Shahzad and Kim (15)	2018	10	10	No	O	4	26–32	3, 1	TP	A	64 Hz	175	TI,MA	LP	MKL-SVM	95.8	88.0	91.7	CV	Yes
Ning et al. (16)	2018	5	4	No	O	10	N/A	6, 4	TP,OH	A,G,M	50 Hz	400	MA	KA	Thresholds	92.0	91.2	90.0	Train set	No
Lee and Tseng (17)	2019	11	4	No	O	4	N/A	4, 0	TP	A	50 Hz	100	MA	N/A	Thresholds	96.0	100	99.0	Train set	No
Salama and Eskaf (18)	2020	19	15	No	P	38	19–75	19, 19	TP,WA	A	50 Hz	1,798	SF,MA	ME	DNN	N/A	N/A	96.1	Test set	Yes

a O, Own; P, Public.

b TP, Trouser's pocket; SP, Shirt's pocket; WA, Waist; BE, Belt; OH, On-hand.

c A, Accelerometer; M, Magnetometer; G, Gyroscope.

d GR, gravity cross-rate; MA, magnitude; TI, tilt; SF, statistical features; VE, velocity; GF, G-force; TD, time domain.

e LP, low-pass; KA, Kalman; ME, mean.

### Publication

**Figure 3:**
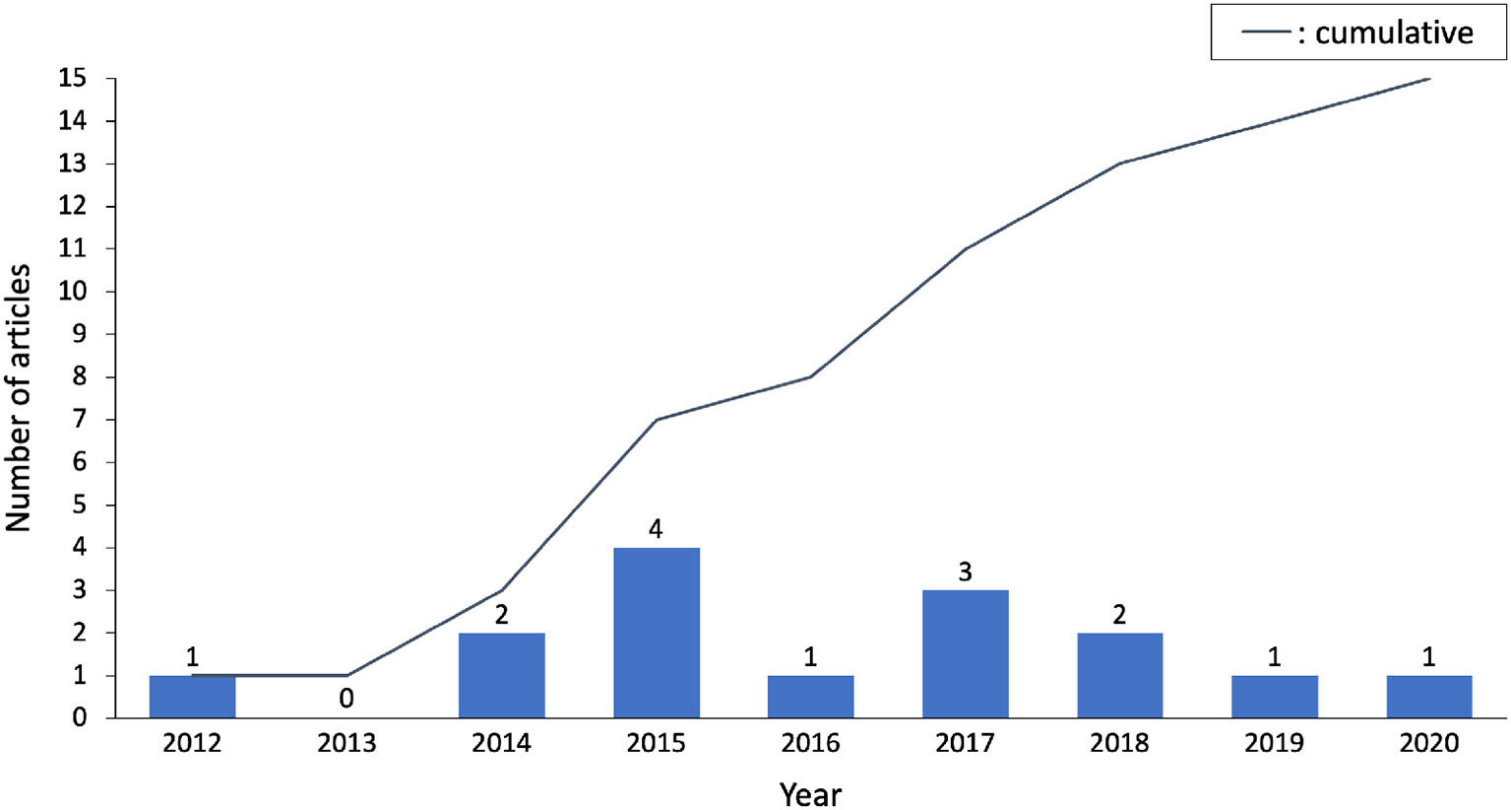
Publication years of selected papers, there is a steady interest for the topic of app-based fall detection.

The selected studies span most of the last decade, ranging from 2012 to 2020. The average and median publication year is 2016, which is in contrast with the concurrent rapid development of data science. It should therefore be kept in mind that earlier studies (4–10) did not have access to same knowledge and tools for their analysis as the most recent ones (13–18). A visualization of the publication years is presented in Figure 3 and highlights a steady interest for the topic.

### Data collection

**Figure 4:**
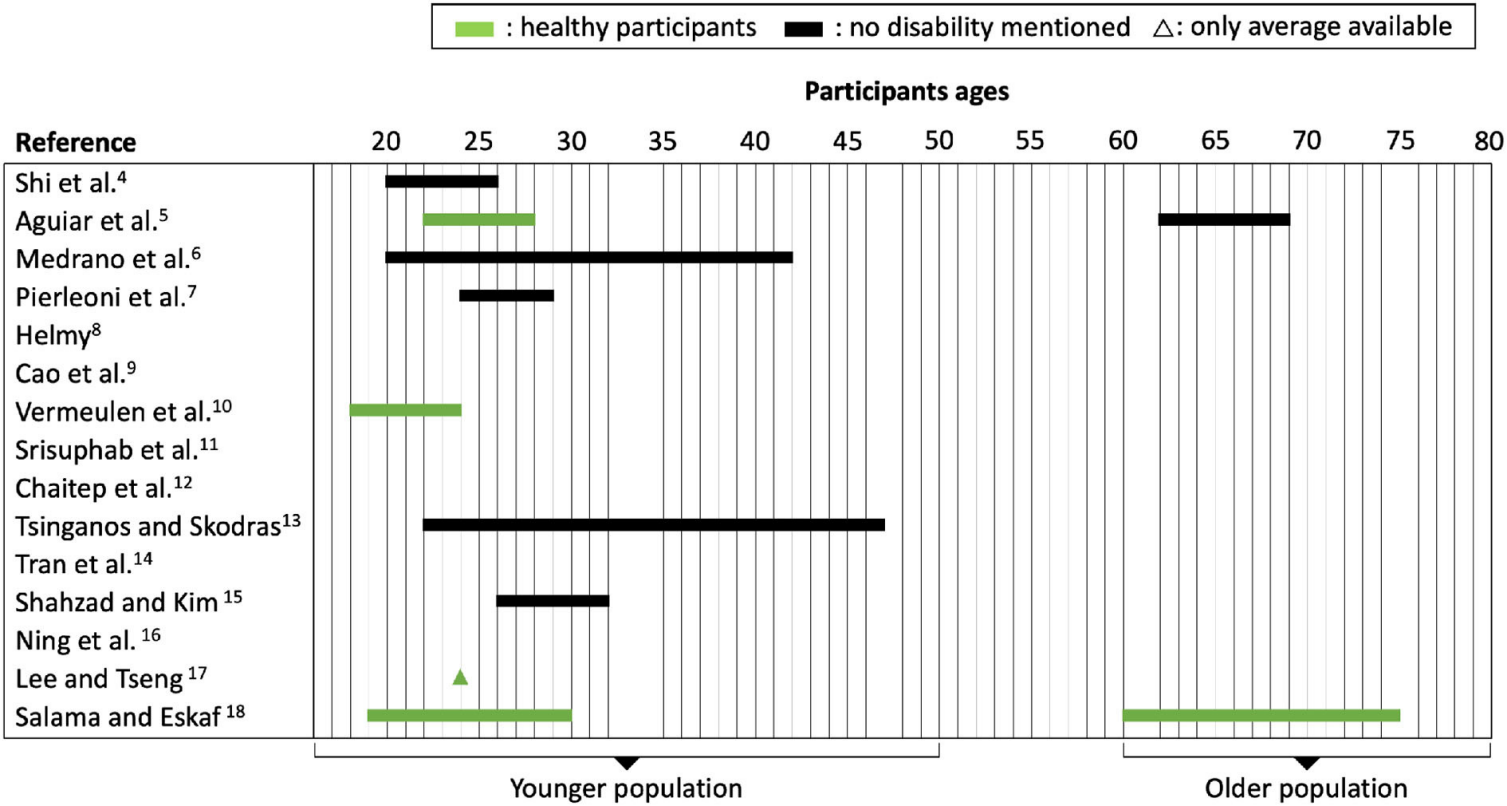
Age range and health condition of participants. Two studies notably included elders who performed Activities of Daily Living tasks.

For data collection, most of the studies (4–12, 14–17) designed their own experimental set-ups and constructed their own fall datasets from scratch. The numbers of participants were relatively low, with an average of 14.8, but this was mostly due to the laborious experimental procedures. Figure 4 displays the age ranges of the participants as well as their reported health conditions. We note in particular that two studies (5, 18) included real data from elderly people, who generally performed only the activities of daily living (ADLs), but not falls. No study mentions having people with disabilities or recovering from an operation in their data. One study (9) had the ingenious idea of adding a dummy with human proportions and weights as a means to simulate difficult falls.

All the selected studies considered the common positioning of phones in pants pockets, although some also investigated a second positioning. In some cases, the researchers were looking for better performance by placing the phone at a higher, yet less common, position on the body, such as the waist (18), belt (10, 14), or shirt pocket (7). In other cases, the researchers focused on additional real-life possibilities, like having the phone in the hand (8, 16) or in a bag (6). Because of the exclusion criteria, all the studies used the accelerometer, with only two (7, 16) leveraging angle sensors as well. Most sampling frequencies (4, 6–8, 10, 13–18) were in the range of 40–60 Hz, with three studies (7, 14, 18) interestingly mentioning that this represents a good trade-off between precision and efficiency. Clearly, with a higher sampling frequency, more data points are collected, but more computational power is then required for both sampling and analysis. One of the articles (9) describes the frequency as a trade-off in precision itself, with an excessively high frequency creating noise and one that is too low missing important signals. Finally, Nyquist's theorem is also mentioned (13), according to which the sampling frequency should be at least double that of the typical 0–20 Hz frequency of human movements.

**Figure 5:**
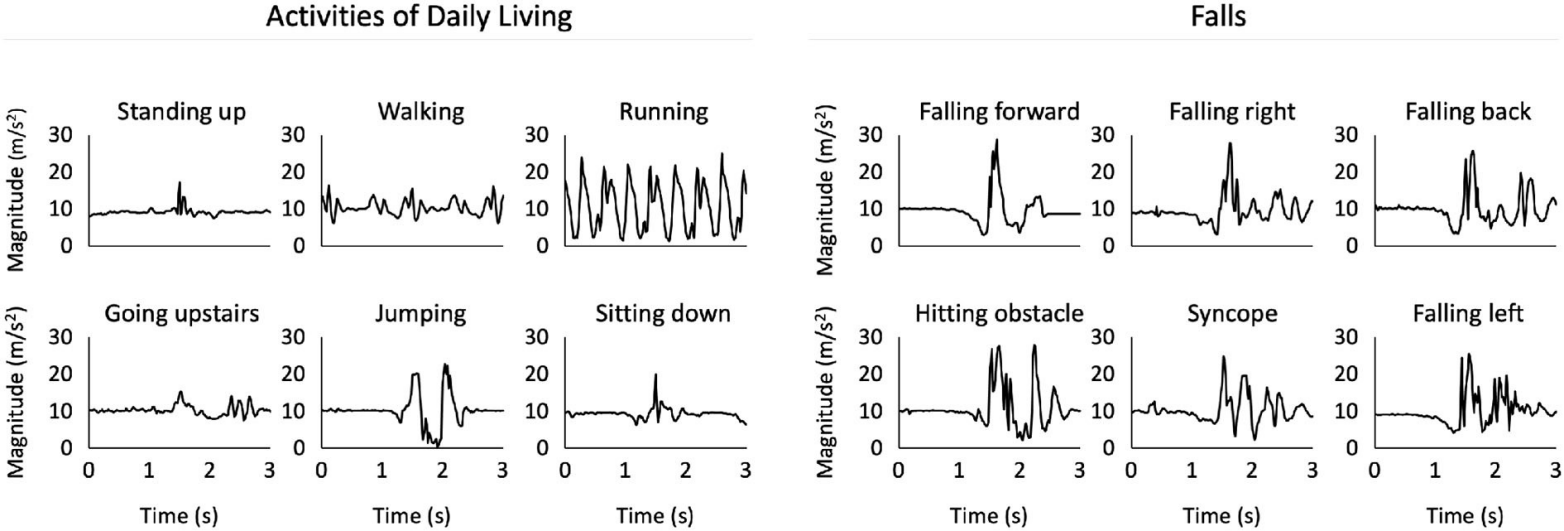
Our plot of acceleration magnitudes for different types of activities of daily living and falls, based on data retrieved from the *UniMiB-SHAR* (25) dataset. Amplitude and signal frequency differences are apparent from visual inspection.

To give the reader a better idea of the signals being recorded, we plotted the acceleration magnitude of ADL and fall samples from the public *UniMiB-SHAR* (25) dataset (Figure 5). The plot is divided into two groups: ADLs and falls. There are clear differences in terms of signal magnitude and frequencies between the two groups. Even inside the ADLs group, distinctive patterns appear based on the activity being performed. Whilst walking and running feature periodic signals, other activities like standing up or jumping lead to a more sudden, one-time acceleration signal. Regarding falls, they all share a sudden rise in acceleration magnitude when the fall occurs and seem to have similar patterns. It is however to keep in mind that the accelerometer records separately the acceleration across the three movement axis (not shown here). Such separation can help classification algorithms distinguish further sub categories of falls.

### Scope

The scope represents the extent to which fall detection was studied. Eleven articles (4, 5, 7, 10, 12–18) reportedly used pre-defined ADLs as negative samples, including activities like walking, sitting, jumping, and walking up and downstairs. While this makes sense from a classification perspective, pre-defining ADLs creates the risk of omitting real-life situations that should be considered to be non-falls. Two articles (6, 9) took the approach of leaving subjects to their normal daily routines and passively recording data while they carried their phones. This latter approach has the advantage of being unbiased regarding the types of daily activities, though it may still miss some rare non-fall events.

Falls themselves were simulated in all cases because recording real unwanted falls would be very challenging due to their rarity. Different fall types were envisioned in all but one study (4–13, 15–18), even though the most dangerous scenarios (like falling down stairs) were never performed, which puts the practicality of the proposed solutions into question as they were not conceived to detect the most perilous types of falls. Finally, the possibility of dropping the phone was only discussed and addressed in three instances (4, 8, 12) despite it being a key event to consider due to its ordinariness and its fall-like pattern.

### Data analysis

**Figure 6:**
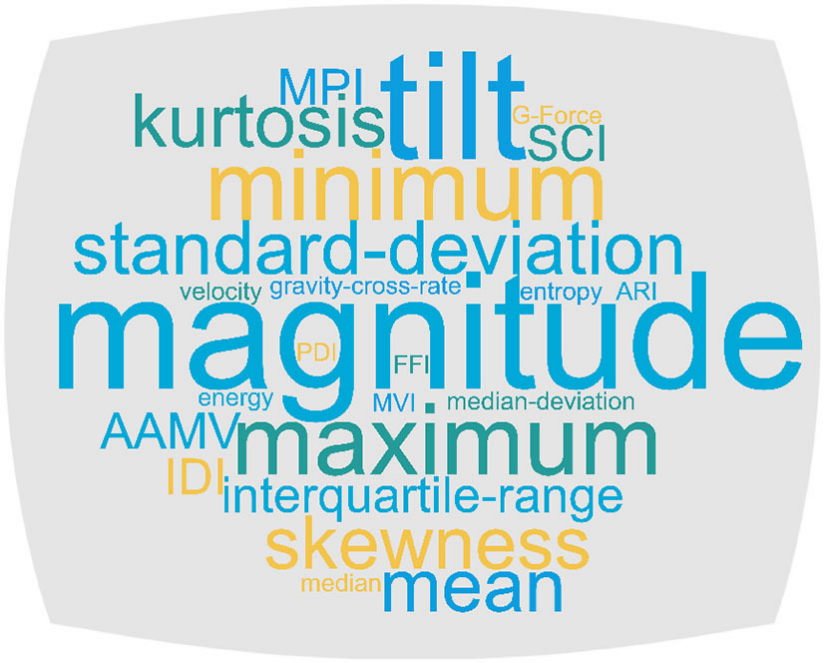
Wordcloud of features engineered in the selected papers. Larger fonts represent higher frequencies, with magnitude and tilt the most prominent.

Feature engineering is often performed after data are gathered from sensors, but before classification. All but one study (4, 5, 7–18) used orientation-invariant features, like magnitude-such orientation-independent methods are preferable to ensure that the user is as unconstrained as possible in their use of the technology. The main features engineered and defined are shown in Figure 6 and explained below. They are based on the acceleration components *a*_*x*_, *a*_*y*_, and *a*_*z*_ as well as the angular velocity components ω_*x*_, ω_*y*_, and ω_*z*_, captured across *N* time samples.

#### Magnitude features

Acceleration magnitude (4, 5, 8–18):

$a_{t}=\sqrt{a_{t,x}^{2}+a_{t,y}^{2}+a_{t,z}^{2}}$

G-Force (12): $G_{t}=\frac{a_{t}}{9.8}$

#### Differential features

Gravity cross-rate (4):

$Z_{g}\;=\;\frac{1}{2N}\overset{N-1}{\underset{i=0}{\sum }}\text{sign}\left(a_{i}-g_{\text{upper bound}}\right)-\text{sign}\left(a_{i-1}-g_{\text{lower bound}}\right)$

Tilt (4, 8, 11): $\cos\theta_{t_{1},t_{2}}=\frac{\vec{a_{t_{1}}}\cdot \vec{a_{t_{2}}}}{a_{t_{1}}a_{t_{2}}}$

Velocity (11): $\vec{v_{t}}=\vec{v_{t_{0}}}+\overset{t}{\underset{i=t_{0}}{\sum }}\vec{a_{i}}dt$

Absolute Acceleration Magnitude Variation (13, 15): $AAMV_{I}=\frac{1}{\left|I\right|}\underset{i\in I}{\sum }|a_{i+1}-a_{i}|$

#### Statistical features

For *k* an axis in *x, y, z*:

Maximum: $M_{k}=\underset{0\leq t\leq N}{\max}\left(a_{t,k}\right)$

Minimum: $m_{k}=\underset{0\leq t\leq N}{\min}\left(a_{t,k}\right)$

Average: $\mu_{k}=\frac{1}{N}\overset{N-1}{\underset{i=0}{\sum }}a_{i,k}$

Standard deviation: $\sigma_{k}=\sqrt{\frac{1}{N-1}\overset{N-1}{\underset{i=0}{\sum }}{\left(a_{i,k}-\mu_{k}\right)}^{2}}$

Skewness: $\mu_{k}^{\left(3\right)}=\frac{1}{N}\overset{N-1}{\underset{i=0}{\sum }}\frac{{\left(a_{i,k}-\mu_{k}\right)}^{3}}{\sigma_{k}^{3}}$

Kurtosis: $\mu_{k}^{\left(4\right)}=\frac{1}{N}\overset{N-1}{\underset{i=0}{\sum }}\frac{{\left(a_{i,k}-\mu_{k}\right)}^{4}}{\sigma_{k}^{4}}$

#### Time domain features

These features are derived from the temporal analysis of a fall event. The following is a brief overview of a few examples; the exact definitions can be found in some of the selected articles (13, 14) and their respective references.

Impact Duration Index: *IDI* = duration of the fall.

Peak Duration Index: *PDI* = duration of the acceleration peak.

First acceleration magnitude value: *FAM* = time of the first acceleration peak above 2*g*.

Peak time: *PT* = time of the peak exceeding the 3*g* threshold.

**Figure 7:**
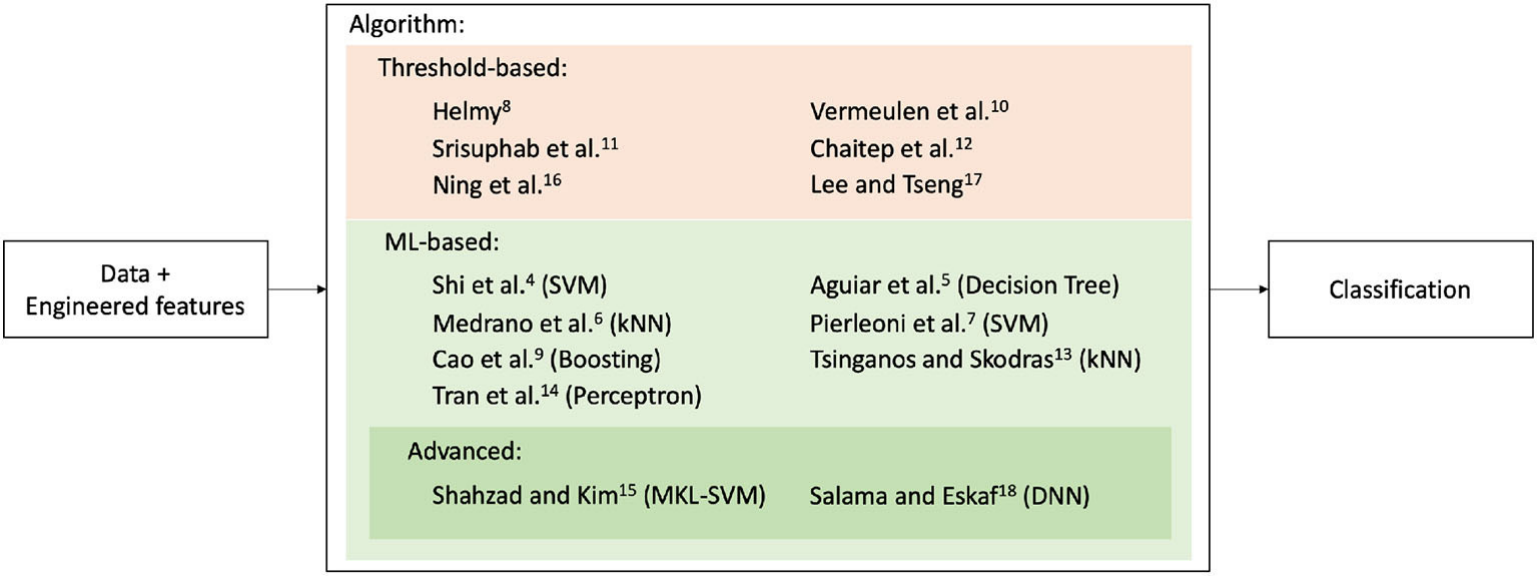
Categorization of algorithms for selected studies between threshold and ML-based. ML-based approaches constitute the majority.

In addition to feature engineering, many of the studies (4, 5, 7, 13, 15, 16, 18) used signal filtering to smooth the signal and remove its highest-frequency components, which represent mostly noise, prior to the detection task. For the classification step, Figure 7 shows a categorization of approaches used between threshold-based and machine-learning-based. Six studies (8, 10–12, 16, 17) used quite basic threshold classifiers, which give a fall alert if a certain quantity (generally derived from the acceleration) exceeds a pre-defined level. While this approach is easily explainable and computationally efficient, it is likely to create many false positives in real-life scenarios. The nine other studies (4–7, 9, 12–14, 18) instead explored machine learning classifiers, with only two (15, 18) going beyond standard machine learning algorithms. The reviewed articles reportedly used the following programming languages for the implementation of their approaches: Java (6–15, 17, 18) (for Android OS), MATLAB (4, 16), and RAPIDMINER (5).

### Evaluation

Commonly reported metrics for evaluating the results are sensitivity, specificity, and accuracy, which are based on the following detection outcomes:

- True Positive (TP): A fall is correctly classified as a fall
- True Negative (TN): A non-fall activity is correctly classified as non-fall activity
- False Positive (FP): A non-fall activity is incorrectly classified as a fall
- False Negative (FN): A fall is incorrectly classified as a non-fall activity

These are defined as follows for binary classification, with *m* the total number of samples:

$\text{Sensitivity}=\frac{\text{TP}}{\text{TP}+\text{FN}}$ $\text{Specificity}=\frac{\text{TN}}{\text{TN}+\text{FP}}$ $\text{Accuracy}=\frac{\text{TP}+\text{TN}}{m}$

**Figure 8:**
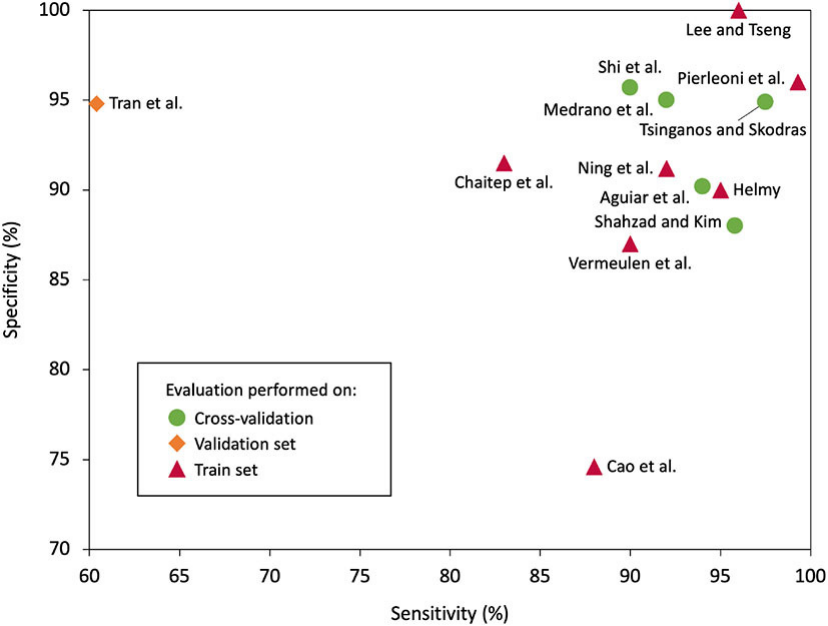
Sensitivity and specificity, if available, for the selected studies. The two best results do not use cross-validation as their evaluation method which may hint at an overestimation of their performances.

Sensitivity is therefore the ability to detect falls when they occur-the higher the sensitivity, the fewer falls are missed. Specificity is the ability to avoid false alarms, with a higher specificity meaning fewer false alarms-one can see a clear trade-off between sensitivity and specificity. Finally, accuracy is a mixture of both, being the ability to correctly classify an event, with no particular focus.At first glance, the studies appear to report high metrics, with an average sensitivity of 90.6%, specificity of 91.5%, and accuracy of 92.2% and the best reaching 99.3% (7), 100.0% (17), and 99.0% (17), respectively. However, these results should be treated with caution for three reasons. First, eight of the articles only used a training set to both tune and evaluate their algorithms, greatly increasing the risk of overfitting and thus producing over-confident results. Figure 8 presents an overview of the reported sensitivities and specificities and contrasts them with the confidence in the evaluation method; it notably shows that the two apparently leading results (7, 17) used an inaccurate evaluation method on the training set. Second, most of the studies used their own original datasets, which are, to different degrees, challenging to classify, depending on the scope of the daily activities considered. Third, all but one11 of the studies evaluated their results offline-that is, the detection app was not running in someone's pocket, but instead used pre-collected data on a computer-which may have caused bias. Among the best approaches to evaluation, we find that the cross-validation by Shi et al. (4) is particularly well-considered because it is the only one that uses the leave-one-subject-out method to properly estimate how their SVM-based classification could perform with new subjects upon which it has not learned.Finally, a thorough evaluation would consider the power consumption of the app, which only six studies reported (5, 8, 9, 13, 15, 18); it should be obvious that a smartphone battery must be able to last for a full day with the app running in the background. Power consumption can be reported using battery percentage per hour (%/h) (5, 8, 15, 18), energy per analysis (J) (9), or electric charge (mAh) (13). The range for those that reported in battery percentage per hour is 0.9–6.0%.

### Bias evaluation and ranking of selected articles

**Figure 9:**
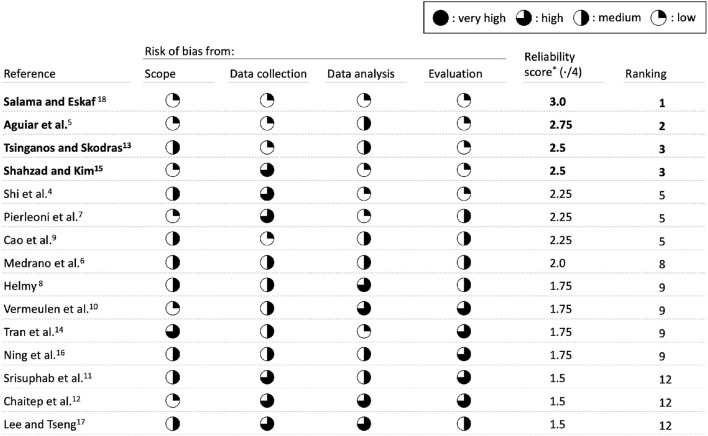
Risk of bias of selected papers across defined dimensions. Four papers stand out due to their quality across several dimensions. *Reliability is given by the number of dimensions (four) minus the risk in each dimension. Each moon quarter increases the risk score of the dimension by 0.25.

Figure 9 summarizes the bias assessment of the approach of each article across the dimensions chosen for the review. Based on the criteria identified in Table 1, we estimated a comparative risk of bias for the 15 selected articles on the following dimensions: scope, data collection, data analysis, and evaluation. The risk score ranges from 0 to 1. After adding the score for each dimension, we can rank each study, as shown in Figure 9. We found Salama and Eskaf (18) to be superior according to our criteria, as they display a low risk of bias across all dimensions. They are followed by Aguiar et al. (5), which also have three dimensions with a low bias risk but a slightly higher risk on the data analysis dimension. This is due to using a decision tree as a classification algorithm, which may lack the complexity to classify tough cases. Tsinganos and Skodras (13) and Shahzad and Kim (15) share third place. Tsinganos and Skodras (13) have a low risk on data collection and evaluation but quite a restrictive scope featuring only four types of falls. In addition, regarding data analysis, they are using kNNs which may be too simplistic in some cases. Shahzad and Kim (15) have a low bias risk on scope, data analysis, and evaluation. Conversely, they feature a high risk on the data collection, as only four participants were included in the data acquisition process, which leads eventually to low statistical significance.

## Discussion

From our findings, we compiled four main areas of improvement for future work on fall detection apps that use smartphone accelerometers.

### Adequation between study scope and end-users habits

Studies must account for the specifics of the population upon which they are focusing in order to replicate them in their experimental investigations. First, regarding smartphone positioning, most of the selected studies (4, 5, 9, 11–13, 15, 17) assumed that the phone would always be placed in the pants pocket. Despite being a common position, this is still a significant assumption that could be erroneous when considering the full span of a normal day. Mechanisms should be implemented to either correctly identify the real position of the device or to encourage the user to put the device where it is expected, such as by sending a notification if the phone is stationary, for instance. Second, the scope of the ADLs considered in a study should be as large-and pre-empt as many false positives-as possible. One can think of many daily actions that might cause a sudden acceleration that could be mistakenly classified as a fall, like lying down or dropping the phone. Such false positives would trigger false alarms in real life, which would deteriorate the user experience and hence the app utilization rate.

### Tailored app design

The needs that the fall app will meet should be clearly identified. Only one study (4) directly asked the targeted population what key functionalities a fall application should offer. It subsequently implemented, for instance, the transmission of relevant personal information alongside the alert. Another study emphasized that, because users may not be tech-savvy, the app interface should be as curated and straightforward as possible and should feature streamlined processes (11). Besides visuals and functionality, battery usage is also critical, and to be truly usable, the smartphone battery should last at least a full day while the app is functioning. This means that battery-saving optimizations should be applied without negatively impacting the app's performance. Among the studies were multiple ideas to minimize power usage, such as leveraging an adaptive sampling frequency (5, 8, 13, 18), avoiding unnecessary computations (15, 18), and using fog computing (9).

### Rigorousness of the data science process

**Figure 10:**
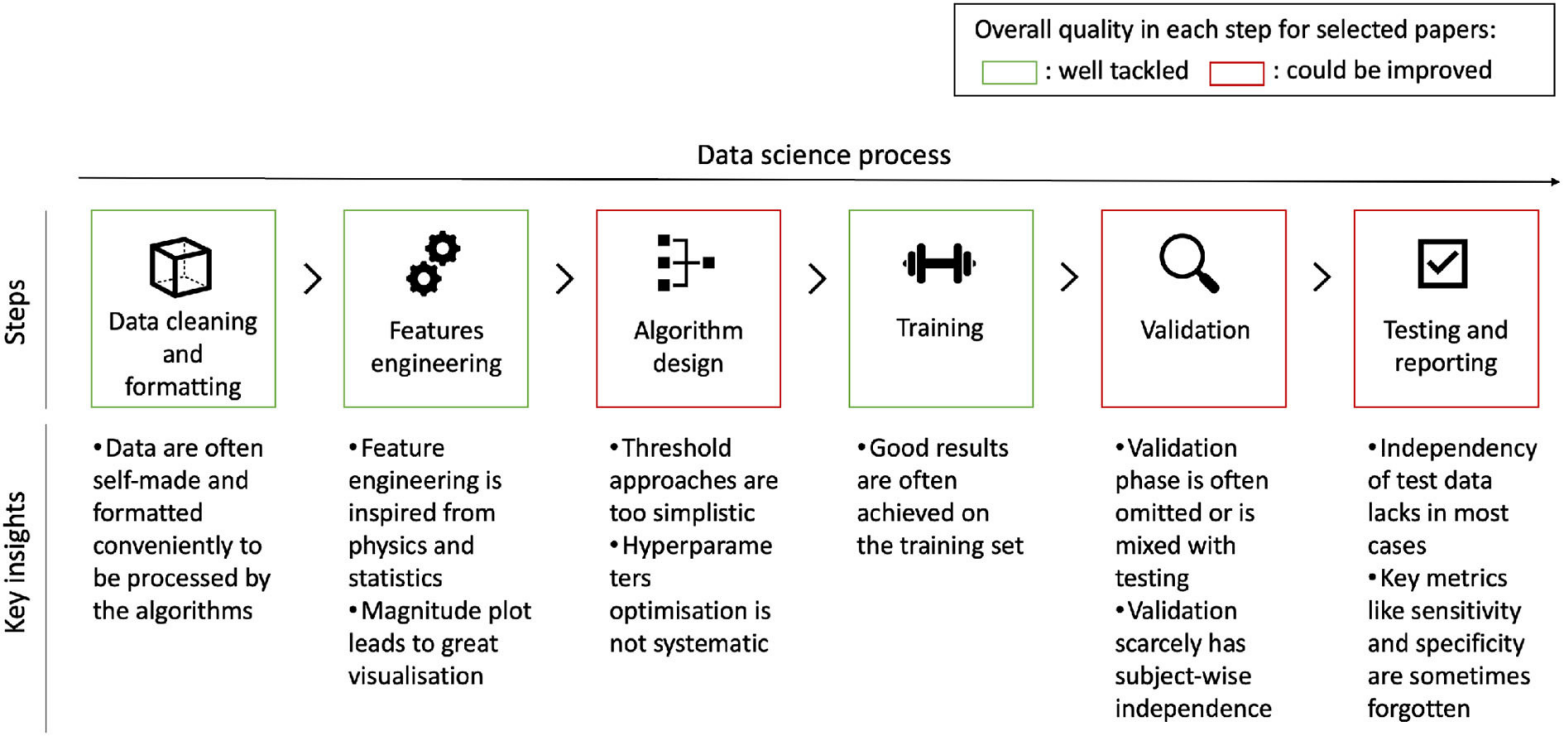
Data science process and overall quality analysis for each step for the included papers. Algorithm design, validation, and testing are notable areas for improvement.

Data science processes should be standardized to ensure both the relevancy and usability of study results. The first area of attention is splitting the dataset into a training set, a validation set, and a test set. A training set allows the algorithm fit to be created, the validation set is used to select hyperparameters, and the test set can then be used for final evaluation and results reporting. If several participants are involved, the data for any given participant should belong only to one of the three sets. Such an approach lays the groundwork for optimization while ensuring that algorithms have not been overfitted and are not reporting overly optimistic results. This is of particular relevance here, given the large number of varieties of fall and of people prone to falling. Figure 10 offers an overview of the overall quality in each step of the process for the selected articles. Another practice that could be systematized is the benchmarking of results. As studies often resort to building their own datasets, comparisons lose their significance. To alleviate this, studies could also report results that they achieve using publicly available datasets. For instance, the datasets *MobiAct* (26) and *UniMiB SHAR* (25) are both recent, public datasets and contain appropriate data for the scope of fall detection studies that the current paper has considered.

### Advanced machine learning opportunities

Advanced machine learning methods are of interest in this research area because of their potential for delivering both good performance and robustness. Besides the two selected studies (14, 18) that employed non-basic machine learning approaches, other studies in the literature (27–29) have considered deep learning methods, and despite not creating apps and thus not being selected for this review, they still laid out interesting ideas. They chose neural architectures that are especially relevant for time series analysis-specifically, gated recurrent units (27) (GRU), long-short term memory networks (28) (LSTM), and convolutional neural networks (29) (CNN). When tested for activity recognition, GRU architecture has notably been shown to outperform LSTM, CNN, support vector machines, Adaboost, and k-nearest neighbors in terms of accuracy and in terms of F1-scores on the publicly available *MobiAct* dataset (27). The better performance of deep learning is generally explained by its ability to account for the temporality of the data compared to traditional machine learning approaches (27). Receiving feedback directly from the user through the reporting of bugs, such as false positives, has also been suggested (28) and could lead to the implementation of both online learning mechanisms and personalization. Further studies should then investigate whether such architectures could be embedded in an app, how they could be used for real-time detection, and whether they lead to excessive power consumption.

### Recommendations for future work

**Figure 11:**
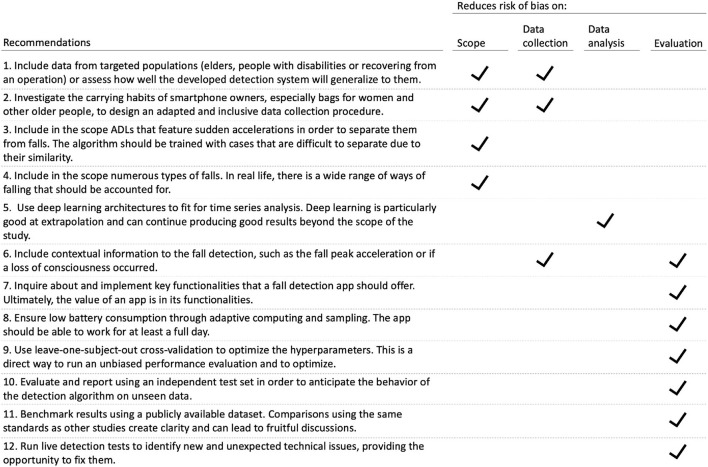
Twelve recommendations to reduce risk of bias on the scope, data collection, data analysis, and evaluation dimensions.

We give in Figure 11 a list of twelve concise recommendations that are considered both actionable and impactful, so that even better fall detection apps with less risk of bias can be built as part of future research.

## Conclusion

In the current review, we first defined relevant criteria for selecting articles closest to having a machine learning-based solution for fall detection. We identified 15 studies with an operating application and defined categories to structure our analysis, notably highlighting narrow scope, uneven depths of data analysis, and possible biases in the evaluation process. We finally constructed 12 actionable and promising recommendations for future studies, which should give more consideration to end-user specifics, data science methodology, and more advanced machine learning.

## Data availability statement

The original contributions presented in the study are included in the article/supplementary material, further inquiries can be directed to the corresponding author.

## Author contributions

ME designed and led the study. TS, ME, RF, and CM conceived the study. All authors approved the final manuscript.

## Funding

Open access funding provided by ETH Zurich.

## Conflict of interest

The authors declare that the research was conducted in the absence of any commercial or financial relationships that could be construed as a potential conflict of interest.

## Publisher's note

All claims expressed in this article are solely those of the authors and do not necessarily represent those of their affiliated organizations, or those of the publisher, the editors and the reviewers. Any product that may be evaluated in this article, or claim that may be made by its manufacturer, is not guaranteed or endorsed by the publisher.
